# CpG Site-Based Signature Predicts Survival of Colorectal Cancer

**DOI:** 10.3390/biomedicines10123163

**Published:** 2022-12-07

**Authors:** Jiande Wu, Lu Zhang, Aditi Kuchi, David Otohinoyi, Chindo Hicks

**Affiliations:** 1 Department of Genetics and the Bioinformatics and Genomics Program, School of Medicine, Louisiana State University Health Sciences Center, Bolivar 533, New Orleans, LA 70112, USA; 2 Department of Public Health Sciences, Clemson University, Clemson, SC 29634, USA

**Keywords:** DNA methylation, signatures pathways, predictive survival, colorectal cancer

## Abstract

Background: A critical unmet medical need in clinical management of colorectal cancer (CRC) pivots around lack of noninvasive and or minimally invasive techniques for early diagnosis and prognostic prediction of clinical outcomes. Because DNA methylation can capture the regulatory landscape of tumors and can be measured in body fluids, it provides unparalleled opportunities for the discovery of early diagnostic and prognostics markers predictive of clinical outcomes. Here we investigated use of DNA methylation for the discovery of potential clinically actionable diagnostic and prognostic markers for predicting survival in CRC. Methods: We analyzed DNA methylation patterns between tumor and control samples to discover signatures of CpG sites and genes associated with CRC and predictive of survival. We conducted functional analysis to identify molecular networks and signaling pathways driving clinical outcomes. Results: We discovered a signature of aberrantly methylated genes associated with CRC and a signature of thirteen (13) CpG sites predictive of survival. We discovered molecular networks and signaling pathways enriched for CpG sites likely to drive clinical outcomes. Conclusions: The investigation revealed that CpG sites can predict survival in CRC and that DNA methylation can capture the regulatory state of tumors through aberrantly methylated molecular networks and signaling pathways.

## 1. Introduction

Despite remarkable progress in screening and patient management, colorectal cancer (CRC) remains a major public health problem [1]. According to the World Health Organization 1,880,725 individuals were newly diagnosed with CRC, representing 9.8% of all newly diagnosed cancers in 2020 [1]. Within the same period, 915,880 individuals died from the disease, representing 9.2% of all cancer-associated deaths [1]. In the United States (US), 147,950 individuals were newly diagnosed with CRC and 53,200 died from the disease in 2020 [2]. Sadly, while the death rate has been declining due to early detection and treatment, the 2021 analysis observed that the number of diagnoses for people under 50 years of age is rising [3]. Most notably, recent reports indicate that worldwide the incidence of CRC is increasing [4,5]. It has been projected that the global burden of CRC is expected to increase by 60% to more than 2.2 million new cases and 1.1 million deaths annually by 2030 [1,5]. Therefore, the need for the discovery of clinically actionable biomarkers and therapeutic targets for CRC cannot be overemphasized.

There is ample evidence from the published reports that lifestyle plays a significant role in the development and progression of CRC [4,5]. At the international level, it has been reported that the patterns and trends in CRC incidence and mortality correlate with present human development levels, and their incremental changes reflect the adoption of more Western lifestyles such as more consumption of processed foods [5]. Given the difficulties in implementing major lifestyle changes or widespread primary prevention strategies to decrease CRC risk, screening has proven to be the most powerful public health tool to reduce mortality [4,5].

A number of screening tests are available for CRC, including colonoscopy; fecal immunochemical test; fecal occult blood test; sigmoidoscopy; multi-target stool DNA (mt-sDNA) test; and computed tomography colonography (CTC) [4,5]. Each of these screening tools has strengths and limitations [4,5]. The quality of evidence supporting these screening tests varies as there have been no head-to-head studies comparing these tools to demonstrate that any one test is superior to another for reducing CRC mortality or incidence [4,5]. Most notably, many screen-eligible people remain unscreened [4,5,6]. It has been reported that approximately one-third of screen-eligible Americans are not screened annually [6,7]. In addition, an estimated 46–63% of deaths from CRC in the United States have been attributed to missed screening opportunities [6,7]. Therefore, there is an urgent need for the discovery of novel clinically actionable molecular diagnostic and prognostic markers to reduce the burden of CRC. A critical unmet medical need in clinical management of CRC pivots around discovery of novel clinically actionable molecular diagnostic and prognostic markers using noninvasive and or minimally invasive techniques for early diagnosis of the disease and prediction of clinical outcomes to reduce the burden of CRC. Because DNA methylation can capture the regulatory state of tumors and can be measured in body fluids, such as urine, blood and stool, it provides unparalleled opportunities for the discovery of novel clinically actionable diagnostic and prognostic markers for predicting clinical outcomes using noninvasive and or minimally invasive techniques.

Advances in microarray and sequencing technologies have enabled comprehensive characterization of CRC genomes and transcriptomes [8,9]. Global genomic projects such as The Cancer Genome Atlas (TCGA) and the International Cancer Genome Consortium (ICGC) have mapped CRC genomes at unprecedented scale [8,9,10,11]. Pan-Cancer Analysis of Whole Genomes (PCAWG) using data from TCGA and the ICGC has provided the most comprehensive catalogues of cancer genomes including CRC [8,9,10,11]. PCAWG has gone beyond previous efforts that focused largely on mapping protein-coding regions of the cancer genomes [11]. In addition, genomic data generated by TCGA and ICGC projects have led to expanded molecular classification of CRC and increased our understanding of the molecular taxonomy of the disease [12,13,14].

Genes may play a strong role in the pathogenesis of CRC, but epidemiologic studies suggest that CRC risk is largely determined by gene and environmental interactions mediated by epigenetic factors [15,16]. Recently, increased attention has focused on understanding the role of DNA methylation because of its influence on the tumor microenvironment during development and progression of CRC [17,18,19]. A recent study based on DNA methylation analysis revealed that enduring epigenetic landmarks can define the tumor microenvironment [19]. Apart from influencing the tumor microenvironment, there are several other properties which have made DNA methylation an attractive area of research focus in CRC [17,18]. DNA methylation can be measured in real time using non-invasive and or minimally invasive techniques in body fluids such as urine, blood and stool [20]. In addition to these properties, methylated DNA fragments have been found to be in abundance in circulating DNA [21,22,23]. These properties have made detection of CpG site methylation in human DNA obtained from urine, blood, stool and in circulating DNA promising approaches for the development of noninvasive and or minimally invasive screening techniques for early diagnosis of CRC [4,5].

However, to date, there has been little information about the ability of DNA methylation or its derivatives, the CpG sites, to predict clinical outcomes such as survival in CRC. This investigation was conducted to comprehensively characterize methylation signatures associated with CRC and to determine whether CpG sites associated with CRC can predict survival. A second, but equally important objective was to discover aberrantly methylated molecular networks and signaling pathways associated with clinical outcomes. Our working hypothesis was that CpG sites associated with CRC can predict survival. We further hypothesized that CpG sites associated with CRC and predictive of survival map to functionally related aberrantly methylated genes interacting in gene regulatory networks and signaling pathways associated with clinical outcomes. We addressed these hypotheses using publicly available DNA methylation data from TCGA generated from tumor samples derived from individuals diagnosed with CRC and matched control samples. For the purposes of this investigation, clinical outcome was defined as either deceased (dead) or alive as reported in TCGA.

The rationale and scientific premise of this investigation was that novel biomarkers such as DNA methylation that could allow and or aid early detection and prediction of clinical outcomes using noninvasive and or minimally invasive methods are of major importance to reducing the burden of CRC. We reasoned that because aberrant DNA methylation is an early event in CRC development, detectable in circulating cell-free DNA and body fluids, and its stable alterations can be easily and rapidly quantified by methylation-specific PCR methods, it provides unprecedented opportunities for the discovery of molecular markers that could aid with early detection of the disease and stratifying patients to guide therapeutic decision making in clinical management of CRC.

## 2. Materials and Methods

### 2.1. Source of DNA Methylation Data and Characteristics of Study Population

We used publicly available DNA methylation data on CRC from TCGA, which has been well catalogued, annotated and linked with clinical information [9]. The methods of data generation and technology platforms used, including experimental protocols, have been well documented by the data originators in TCGA [9]. Here, we provide a brief but detailed description of the source of data, methods of data processing, analysis and integration strategies used.

DNA methylation (array-based) data, and clinical information were obtained from TCGA [24]. The data were downloaded from the Genomics Data Commons data portal using the data transfer tool [25]. DNA methylation was generated using the Illumina HumanMethylation450 BeadChip, containing 485,578 probes [26,27]. The platform has been widely used for quantifying DNA methylation and has been successfully validated [26,27]. The original data set included a total of 466 CRC samples. We processed the samples linking molecular data with clinical information. Clinical information included: sample ID, age at diagnosis, primary diagnosis, race/ethnicity, stage, days to death, days to last follow up, and dead or alive as provided in TCGA clinical file. After linking molecular data with clinical information, we identified 37 individuals with both tumor and adjacent non-tumor control tissue samples (i.e., *n* = 37 tumors and *n* = 37 matched controls) with long-term follow up, which we used in downstream analysis to identify a signature of differentially methylated CpG sites associated with CRC and a signature of CpG sites predictive of survival. The use of only 37 individuals with both tumor and adjacent non-tumor control tissue samples was necessitated by the understanding that DNA methylation can be population-specific. Thus, drawing comparison samples from the same patient group can effectively eliminate confounding factors due to patient differences.

### 2.2. Bioinformatics and Statistical Analysis of DNA Methylation Data

We processed and checked the data for quality using the R package MINFI [28,29]. Poor-performing probes were filtered out prior to differential methylation analysis. This QC step was undertaken because the signals from these probes were unreliable and could confound the analysis, leading to erroneous findings. During the QC step, probes were removed if they met the following criteria: (i) probes that had failed in one or more samples; (ii) probes from the X and Y chromosomes [29]; (iii) probes that were known to have common SNPs at the CpG sites [29]; (iv) probes affected by SNPs, or only those with minor allele frequencies greater than a specified value [29]; (v) probes that were methylated or unmethylated in all samples; (vi) probes that had shown to be cross-reactive. This filtering approach has been successfully used in DNA methylation data analysis [29,30]. To minimize unwanted variation within and between samples, we normalized the data using the stratified Quantile normalization method which considers different probe types [28,29,30]. To examine the distribution properties and dispersion of the data, we applied principal component analysis (PCA), an unsupervised method to investigate variation, the similarities and the differences between the two sample groups.

Subsequently, using normalized data, we computed the detection *p*-value by comparing methylation profiles between tumor and control samples using the MINFI method [28,29,30]. We generated a detection *p*-value for each CpG site in each sample, which represented the quality of the signal. The approach calculates the detection *p*-value by comparing the total signal (M + U) of each CpG site to the background signal level estimated from the negative control probe thereby adjusting for background noise [28,29]. Under this approach, very small *p*-values indicate reliable signals, while larger *p*-values indicate poorer quality signals. We computed the differences between each sample type and averaged them across samples to determine whether there were overall significant differences in mean methylation levels for each CpG site. We performed this analysis on a matrix of β-values, obtaining a moderate t-statistic and associated *p*-value for each CpG site. Because the analysis involved performing a large number of hypothesis tests, we employed the false discovery rate (FDR) procedure [31] to compute *p*-values corrected for multiple hypothesis testing. The discovered CpG sites were annotated for gene names, positions, DNA methylated regions (DMRS) and ranked on adjusted *p*-values. We used a Volcano plot to select CpG sites based on differential methylation value calculated as mean (β_tumor_) − mean (β_normal_), combined with adjusted *p* values. We performed unsupervised analysis using hierarchical clustering to group aberrantly methylated genes and samples together based on the similarity of their patterns of methylation profiles and the direction of change defined as hypermethylated if up- or hypomethylated if down-regulated. This approach was used to identify genes that were co-regulated and associated with CRC. Due to the small sample sizes, we did not stratify samples by age or ethnicity. However, a quantitative assessment of CpG sites was conducted and correlated with clinical information, specifically dead or alive and tumor stage.

Having identified the CpG sites associated with CRC, we conducted analysis to discover a prognostic signature and to determine whether the CpG sites associated with CRC could predict survival. To address this need, we compared the patterns of DNA methylation between patients who survived and patients who died from CRC using information on CpG sites associated with the disease. Here, we sought to discover novel prognostic markers correlated with different survival indicators for individualized prognosis prediction for CRC patients. Differential methylation patterns between dead versus alive patient groups was achieved by computing the median of beta values for the CpG sites associated with CRC. We categorized patients into high-methylated (up) or low-methylated (down) groups based on probe level DNA methylation. Survival analysis was conducted based on time to death data obtained from clinical information provided in TCGA. We performed survival analysis by comparing the median beta values of DNA methylation levels between the dead and alive groups, using information on differentially methylated CpG sites. Survival was computed as a probability score indicating whether the patient was dead or still alive at a particular time point. Using information on survival and time to death, for each time point, we calculated the number of deaths observed in each group, and the expected number if there were in fact no differences between the two groups.

To correlate the information on differentially methylated CpG site level with the patient’s survival, we used the Kaplan–Meier estimator of the survival curve, a tool for visualizing the results of methylation level that involves a time to event analysis [32]. To detect a significance difference between methylation level groups when the risk of death is consistently greater for one group than another, we employed the log-rank test [33]. We used the log-rank test to test the null hypothesis that there is no difference in the probability of death between populations at any time point [32,33]. The log-rank test is the most popular and reliable method for comparing the survival of groups [33]. This approach takes the whole follow-up period into account when predicting survival [33].

### 2.3. Functional Network and Pathway Analysis

To gain insights about the broader biological context in which the aberrantly methylated CpG sites and genes associated with CRC and predictive of survival operate, we performed network and pathway analysis using the Ingenuity Pathway Analysis (IPA) software [34]. Our working hypothesis was that aberrantly methylated genes and CpG sites associated with CRC and predictive of outcomes are functionally related and interact in gene regulatory networks and signaling pathways, which in turn drive the disease and clinical outcomes. We mapped aberrantly methylated genes containing the most significantly highly differentially methylated CpG sites associated with the disease, which included the genes containing CpG sites predictive of survival, onto gene networks and canonical pathways using IPA [34]. We computed the Z-scores to identify aberrantly methylated gene networks with the highest scores associated with CRC and survival, and the *p*-values to determine the probability of correctly assigning aberrantly methylated genes to the correct networks, functional categories and disease type. Aberrantly methylated gene networks were ranked on Z-scores and predicted functional categories were ranked on *p*-values. For pathway prediction, we computed the *p*-values using Fisher’s exact t-test and the FDR to determine the probability of correctly assigning aberrantly methylated genes and associated CpG sites to signaling pathways. The pathways were ranked based on log *p*-values. We performed Gene Ontology (GO) analysis [35] implemented in IPA to determine the molecular function, biological process and or cellular component in which the aberrantly methylated genes and CpG sites associated with CRC and predictive of outcome are involved.

## 3. Results

Despite remarkable progress in patient management, CRC remains a major public health problem worldwide [1,2,3,4,5]. While optimal population-based cancer screening methods have been relatively effective, the incidence of CRC continue to rise unabated. Because CRC detection at early stages increases the prospects of successful and curative treatment, leading to lower incidence of recurrences, and because current parameters for cancer patients’ stratification have been associated with varying outcomes, there is an urgent need for the discovery of molecular diagnostic markers for early detection of the disease and prognostic markers for patient stratification to guide therapeutic decision making in clinical management of CRC. This investigation was conducted to address this critical unmet need by analyzing differential methylation patterns genome-wide between tumor samples and matched controls to comprehensively characterize the patterns of DNA methylation and discover a signature of genes and CpG sites associated with CRC, a signature of CpG sites that were predictive of survival, and aberrantly methylated molecular networks and signaling pathways likely to drive clinical outcomes. In this section and accompanying subsections, we present the results of the investigation.

### 3.1. Assessing Variability and Differences in Patterns of DNA Methylation Profiles

**Figure 1:**
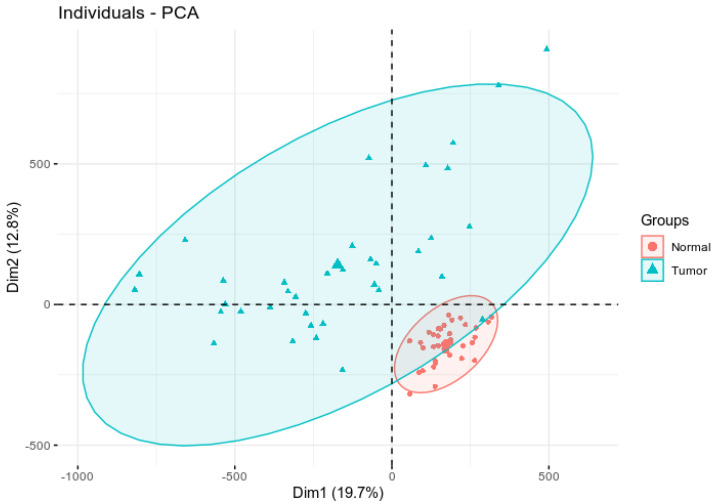
Principal component analysis plot showing differential clustering of tumor vs. control samples. The *x* axis represents the most variation in the data and the *y* axis represents the second most variation in the data.

We performed dimension reduction of DNA methylation data using PCA analysis to identify aberrantly methylated genes and CpG sites separating CRC tumor samples from controls. Here we sought to investigate whether there is significant variation in patterns of DNA methylation profiles within and between tumor samples and matched controls, and whether employing PCA could capture the variations distinguishing tumor from control samples. The results of this investigation are presented in (Figure 1). As shown in Figure 1, the first principal component clearly separated tumor samples from control samples indicating differences in epigenomic alterations between cases and controls. The normal samples were clustered together, while the tumor samples exhibited significant variation in patterns of methylation profiles. Overall, principal component one captured the largest source of variation in the data with one outlier, whereas dimension two captured the second largest source of variation in the data (Figure 1). The observed variation within tumor samples can partially be explained by the mixture of tumor samples which was detected by correlating tumor stage with patterns of DNA methylation. In that assessment, tumor samples at different stages of diagnosis exhibited variation in patterns of DNA methylation, with some being well-differentiated, while others were not. Under such conditions, the observed outcome in Figure 1 was expected. Interestingly, despite the significant variation within tumor samples, dimensional reduction using PCA indicated that DNA methylation data contains variability that can distinguish tumors from control samples (Figure 1). The significant variation in tumor samples suggested that individuals diagnosed with CRC may have different prognoses and may be amenable for DNA methylation-based patient stratification. This observation formed the foundation to test the hypothesis that CpG sites associated with CRC could potentially be used to predict survival, reported in Section 3.3.

### 3.2. Discovery of DNA Methylation Signatures Associated with CRC

One of the primary objectives of this investigation was to discover and characterize a DNA methylation signature and regions associated with CRC. To address this issue, we tested the hypothesis that there are significant differences in patterns of DNA methylation profiles between tumor and control samples, by comparing the pattern of DNA methylation between the two sample groups as described in the methods section. Here we sought to identify a signature of CpG sites and genes associated with CRC, which could be used in downstream analysis to identify CpG sites and genes predictive of survival outcomes.

Comparison of patterns of DNA methylation between tumor and matched controls samples produced 117,026 significantly differentially (*p* value < 0.05) methylated CpG sites located in DNA methylated regions (DMRs) distributed widely throughout the methylome and mapped in or near 16,733 known genes. Out of all the aberrantly methylated genes discovered, 3097 genes contained a single significant CpG site, whereas 13,636 genes contained two or more significant CpG sites, indicating that DNA methylation is highly prevalent in tumor genomes. When we increased the threshold to a *p*-value cutoff of 10^−15^, we discovered 779 highly significantly aberrantly methylated CpG sites mapped to 464 genes. Among the 779 most highly differentially methylated CPG sites discovered, 271 CpG sites mapped to 202 genes and were hypermethylated, whereas 508 CpG sites mapped to 262 genes and were hypomethylated. This suggests that hypomethylation may be more prevalent in tumors than hypermethylation, which is consistent with results from a previous report [19].

The results showing the top 50 CpG sites which were most highly significantly associated with CRC and the genes they map to are presented in Table 1. Also presented in the Table are the DMRs in which the discovered CpG sites are located. To determine whether any of the most highly methylated genes discovered have been implicated in CRC, we performed in silico validation using published reports. Among the genes containing the top CpG sites shown in Table 1 are the genes *ADAMTS2*, *ADAMTS5*, *ADARB2*, *ADCY1*, *ADHFE1*, *AGRN*, *AKR1B1*, *ANK1*, *ANKRD13B*, *ANXA2*, *AQP5*, *ATP11A*, *BCAT1*, *BEND5*, *BOLL*, *CADM2* and *CASR*, which have been implicated in CRC [36,37,38,39,40,41,42,43,44,45,46,47,48,49,50,51,52]. A complete list of all CpG sites significantly associated with CRC and the genes they map to, including DMRS in which they are located, is presented in Supplementary Table S1. Overall, the investigation confirmed our hypothesis that there are significant differences in the patterns of DNA methylation profiles between tumor and control samples, suggesting that DNA methylation profiling of tumor and control samples could lead to measurable changes that could potentially guide therapeutic decision making in clinical management of CRC.

**Figure 2:**
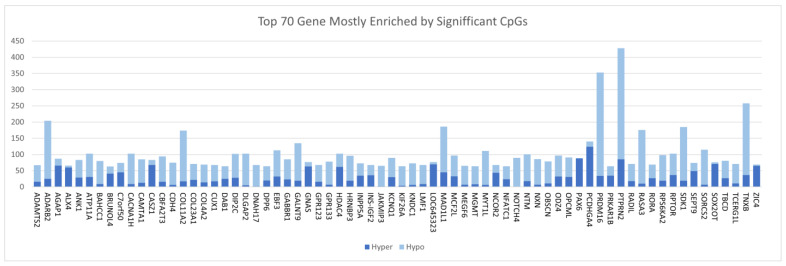
The 70 most highly methylated genes. The number of methylation sites is plotted on the *y* axis and the gene names are plotted on the *x* axis.

Having discovered CpG sites and genes associated with CRC, we computed the frequency of CpG sites per gene to identify a signature of the most aberrantly methylated genes and the type of methylation, whether hyper (up) or hypo (down). The genes were ranked based on the number of CpG sites significantly associated with CRC. The investigation revealed extensive CpG sites mapped to a wide range of DMRs associated with CRC. The results showing the 70 most highly significantly differently methylated genes containing both hypermethylated and hypomethylated regions in the tumor genome compared to the control group are shown in Figure 2. The DMRs included both hypermethylation and hypomethylation events associated with CRC (Figure 2). As shown in Figure 2, hypomethylated events were more prevalent that hypermethylation events. A complete list of all genes containing hypermethylated and hypomethylated regions is presented in Supplementary Table S1. The high prevalence of hypomethylation events suggests that epigenetic silencing may play a role in CRC.

To determine the similarity and dissimilarity in patterns of DNA methylation profiles for the top CpG sites that were most highly significantly associated with CRC, we performed a two-way hierarchical clustering using beta values. Here we sought to discover clusters of CpG sites with similar patterns of DNA methylation in the tumor samples and controls by addressing the hypothesis that genes containing CpG sites associated with CRC are likely co-regulated and have similar patterns of DNA methylation in tumor and control samples. For this analysis, we used the 779 CpG sites and 446 genes that were most highly associated with CRC, as these were the most informative and could eliminate spurious patterns.

**Figure 3:**
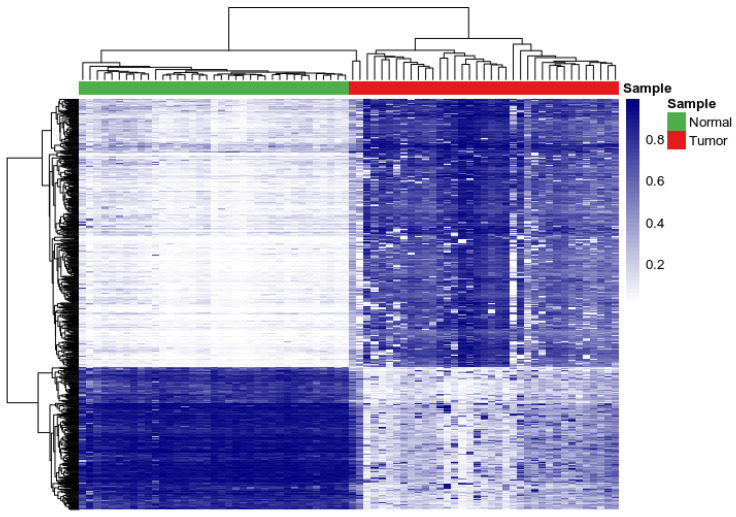
Patterns of DNA methylation for tumor and normal paired samples for the top 779 most highly significantly differentially methylated CpG sites between tumor and normal samples associated with CRC. Blue color indicates high beta value and white color indicates low beta value.

The results of hierarchical clustering are presented in a heatmap in Figure 3. We discovered differences in patterns of DNA methylation between tumor samples and controls. In one cluster, genes containing CpG sites with lower beta values in controls had higher beta values in tumors (Figure 3). In the other cluster genes containing CpG sites with higher beta values in controls had lower beta values in tumors (Figure 3). A complete list of all the 779 CpG sites and the genes they map to is presented in Supplementary Table S2.

### 3.3. Discovery of A Prognostic Signature and Survival Prediction

Another objective of this investigation was to determine whether CpG sites associated with CRC could predict survival. Thus, having discovered and characterized the CpG sites associated with CRC, we performed a survival analysis using the information on the most significant CpG sites. In this approach, patients were regrouped for different CpG sites using the beta value of each probe. Samples with beta values greater than the median value were considered the hypermethylated group, and samples with beta values below the median value were considered the hypomethylated group. Out of a total of 37 patients in the study, 36 (97.3%) patients had died at the last follow-up visit. For each probed CpG site, patients were clustered in different groups and survival was computed as the probability of that individual’s survival up to a particular time point as described in the methods section. We performed Kaplan–Meier analysis and a log-rank test for each probe to evaluate its association with overall survival in the set as described in the methods section.

**Figure 4:**
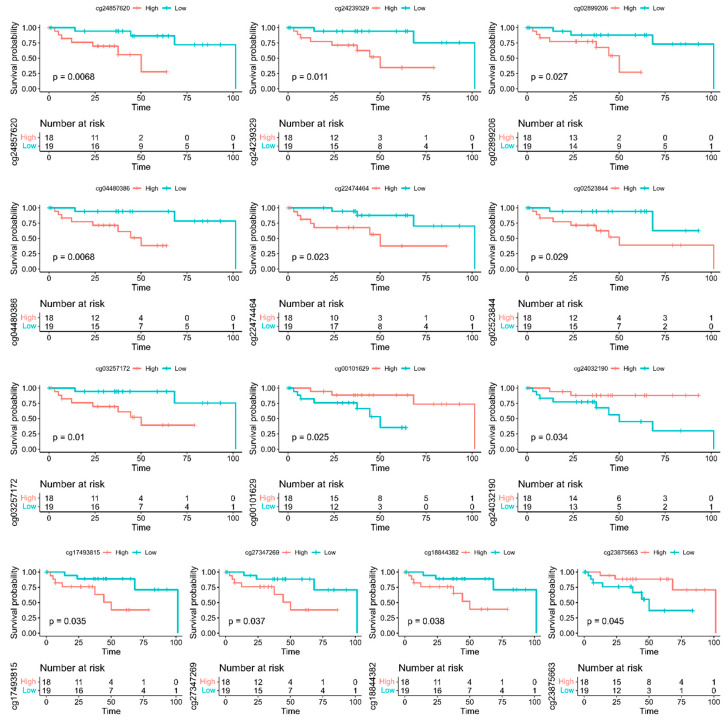
Kaplan–Meier survival analysis of clusters of samples defined by 13 CpG sites, time expressed in months. Thirteen CpG loci with *p*-value < 0.05 were identified, including *cg24857620(NPBWR1)*, *cg04480386(CDH12)*, *cg03257172(NR5A2)*, *cg24239329(DCLK1)*, *cg22474464(NKX2-2)*, *cg00101629(KIAA1026)*, *cg02899206(ADARB2)*, *cg02523844(MAGI2)*, *cg24032190(SMAD3)*, *cg17493815(GUCY1B3)*, *cg27347269(DOK6)*, *cg18844382 (EFS*) and *cg23875663(PCSK2)*. High is hypermethylation and low is hypomethylation. Number of individuals at risk is shown in table below each Kaplan–Meier survival curve plotted as a function of time.

The results showing the most significantly differentially methylated CpG sites between the two sample groups (dead vs. alive) are shown in Table 2. Also shown in the Table are the genes to which the CpG sites map, DMRs and estimated *p*-values. We discovered 13 CpG sites (*p* < 0.05) mapped to 13 genes (*NPBWR1*, *CDH12*, *NR5A2*), *DCLK1*, *NKX2-2*, *KIAA1026*, *ADARB2*, *MAGI2*, *SMAD3*, *GUCY1B3*, *DOK6*, *EFS* and *PCSK2*) that were predictive of survival (Table 2). Figure 4 shows the results of survival analysis for the 13 CpG sites that were predictive of survival along with probe IDs. In the figure, the names of the genes the CpG sites map to are included in the Figure legend. To determine whether any of the discovered genes containing CpG sites predictive of survival have been implicated in CRC, we performed in silico validation using published literature. In silico validation revealed the genes *CDH12*, *NR5A2*, *DCLK1*, *NKX2-2*, *ADARB2*, *MAGI2*, *SMAD3*, *GUCY1B3* and *EFS* (Figure 4), which have been implicated in CRC [38,52,53,54,55].

The results showing Kaplan–Meier curves displaying the estimated survival probability for two different groups of patients with different methylated CpG sites are presented in Figure 4. Each vertical step in the curve indicates one or more deaths. Right-censored patients are indicated by a vertical mark in the curve at the censoring time (month). The log-rank test indicates a significant difference between the survival curves. A visual inspection suggests that ten (10) hypomethylated (low methylated) CpG sites (shown in Table 2) had significantly better overall survival rates than the three (3) hypermethylated (high methylated) CpG sites. Overall, three hypermethylated (high methylated) CpG sites (shown in Table 2) had significantly better survival rates than the hypomethylated (low methylated) CpG sites.

### 3.4. Discovery of Aberrantly Methylated Molecular Networks and Signaling Pathways

A secondary but equally important objective was to discover aberrantly methylated gene regulatory networks and signaling pathways and their functional connectivity. Here we sought to gain insights about the broader biological context in which the CpG sites and aberrantly methylated genes associated with CRC and predictive of survival operate, and to discover the molecular networks and signaling pathways they control. To achieve this objective, we performed network and pathway analysis using IPA as described in the methods section. For this analysis we used the 464 genes containing the 779 CpG sites that were most highly significantly associated with CRC, which included the 13 genes containing the 13 CpG sites predictive of survival.

Network analysis produced 25 aberrantly methylated molecular networks with Z-scores ranging from 11 to 47. The molecular networks were enriched for CpG sites mapped to functionally related aberrantly methylated genes with overlapping functions. The top seven networks with Z-scores ranging from 31 to 47 contained aberrantly methylated genes predicted to be involved in cancer, gastrointestinal disease, organismal injury and abnormalities, organismal development, tissue development, cellular development, cellular growth and proliferation, and metabolic disease. In addition, the analysis produced aberrantly methylated networks containing genes predicted to be involved in nucleic acid metabolism, small molecule biochemistry, cell signaling, cell-to-cell signaling and interaction, molecular transport, infectious diseases, cellular assembly and organization, cell morphology, inflammatory response, endocrine system disorders, amino acid metabolism, energy production, post-translational modification, cell death and survival, digestive system development and function, antimicrobial response, connective tissue development and function, cellular function and maintenance, and inflammatory response. A complete list of molecular networks enriched for CpG sites and functionally related aberrantly methylated genes they map to, as well as information on top diseases and molecular functions they are involved in, is contained in Supplementary Table S3. 

Pathway analysis revealed 284 signaling pathways enriched for CpG sites mapped to functionally related aberrantly methylated genes associated with CRC. Among the top signaling pathways discovered include synaptogenesis, circadian rhythm, corticotropin releasing hormone, CREB, GI, synaptic long term depression, G-Protein coupled receptor, cAMP-mediated, endocannabinoid cancer inhibition, dopamine-DARPP32 feedback in cAMP, white adipose tissue browning, netrin signaling, dopamine receptor, endocannabinoid neuronal synapse, ephrin receptor, cardiac Î^2^-adrenergic, endocannabinoid developing neuron, glutamate receptor, dermatan sulfate biosynthesis and axonal guidance signaling pathways. A complete list of all the significant pathways enriched for CpG sites is presented in Supplementary Table S4.

Overall, the results from functional analysis revealed functionally related aberrantly methylated genes with overlapping functions, interacting in gene regulatory networks and signaling pathways. Most notably, the 13 genes containing 13 CpG sites associated with CRC and predictive of survival were found to interact with other genes containing CpG sites highly significantly associated with CRC, suggesting that genes containing CpG sites predictive of survival may be regulated by or may be regulating other genes. Thus, the investigation addresses the two objectives by (1) showing that CpG sites associated with CRC can predict survival, and (2) revealing aberrantly methylated molecular networks and signaling pathways associated with clinical outcomes.

## 4. Discussion

A critical unmet medical need in clinical management of CRC pivots around lack of noninvasive and or minimally invasive techniques for early detection of the disease and lack of molecular markers for stratification of patients to guide decisions in clinical management of the disease. To address this knowledge gap and critical unmet medical need, we analyzed the patterns of DNA methylation to discover signatures of CpG sites associated with CRC and that could predict survival, and network states and signaling pathways they control. The investigations revealed CpG sites mapped to extensive hypermethylated and hypomethylated DMRs associated with CRC. We discovered 13 CpG sites associated with CRC that accurately predicted survival outcomes. In addition, we discovered aberrantly methylated gene regulatory networks and signaling pathways associated with clinical outcomes. The results associating DNA methylation with CRC discovered in this report are consistent with the results from previous reports [53,54]. The novel and innovative aspect of our investigation is that CpG sites associated with CRC not only have the promise to serve as potential molecular markers for early detection of the disease, but could potentially also be used for stratifying patients to guide therapeutic decision making in clinical management of the disease. The discovery of aberrantly methylated signaling pathways suggests that DNA methylation holds the promise for the discovery of therapeutic targets.

The clinical significance of our investigation is that DNA methylation-based molecular markers have the promise for detection and clinical management of CRC. Although the DNA methylation biomarkers we discovered were not clinically validated, which could preclude their implementation in clinical practice, in silico validation revealed important aberrantly methylated genes including *NPBWR1*, *CDH12*, *NR5A2*, *DCLK1*, *NKX2-2*, *KIAA1026*, *ADARB2*, *MAGI2*, *SMAD3*, *GUCY1B3*, *DOK6*, *EFS* and *PCSK2* implicated in CRC [32,33,34,35,36,37,38,39,40,41,42,43,44,45,46,47,48,49,50,51,52,53,54,55], suggesting that if further confirmed these genes could serve as prognostic markers. For example, the cadherin-12 (*CDH12*) gene has been shown to increase cancer cell migration, invasion and progression via promoting EMT by targeting the transcriptional factor Snail [52,56]. *CDH12* has also been shown to be a predictor of prognosis in CRC patients and as an oncogene in CRC cell proliferation [52,56]. It has been shown that down-regulation of *NR5A2* is linked to worse overall survival of CRC patients [57]. The *DCLK1* gene is reported to be up-regulated and associated with metastasis and prognosis in CRC [58]. Cytoplasmic expression of DCLK1-S, a novel DCLK1 isoform, has been shown to be associated with tumor aggressiveness and worse disease-specific survival in CRC [59]. The NKX2.2 gene is known to be a tumor suppressor in CRC due to hypermethylation [55]. A recent study on CRC revealed that overexpression of the NKX2.2 gene suppressed cell proliferation, colony formation, and inhibited tumor invasion and migration in CRC cells [55]. There is ample evidence that SMAD3 genetic variants can predict overall survival in metastatic CRC for patients treated with FOLFIRI therapy [60]. Taken together these observations support the hypothesis that DNA methylation-based molecular markers discovered in this investigation have potential to serve as clinically actionable biomarkers for early detection of CRC and predicting outcome.

Network analysis revealed molecular networks enriched for CpG sites mapped to aberrantly methylated functionally related genes predicted to be involved in cancer, gastrointestinal disease, cellular development, cellular growth and proliferation, inflammation, and metabolic disease, all of which are highly relevant to the pathogenesis, development and progression of CRC [61,62,63,64]. For example, disruption of the gut microbiota has been linked to gastrointestinal disease, which causes CRC [61]. The link between inflammation and CRC has been reported [64]. It has been reported that inflammation-related biomarkers could be used to predict prognosis in CRC [64]. Metabolic syndrome has been associated with an increased risk of CRC incidence and cancer-specific mortality [63]. Recently, there has been speculation that the observed rapid increase in CRC incidence among individuals under the age of 50 over the last 20 years may be linked to metabolic syndrome and obesity [65,66,67].

The discovery of aberrantly methylated signaling pathways including the circadian rhythm, CREB, GI, G-Protein coupled receptor, cAMP-mediated, endocannabinoid cancer inhibition, white adipose tissue browning, dopamine receptor, ephrin receptor, glutamate receptor and axonal guidance signaling pathways implicated in CRC suggests that DNA methylation has promise for the discovery of potential therapeutic targets. Circadian clock genes discovered here are reported to be involved in cell cycle regulation, oncogenesis, tumor suppression, DNA repair, cell proliferation, cell death and cell growth [68]. The clock-controlled genes regulate the timing of cellular basic functions such as metabolism, DNA damage repair, and autophagy, all of which play a role in CRC [69,70]. There is ample evidence from the published literature that the circadian system regulates cell growth and death by affecting transcription/post-translational modification of critical proteins for DNA replication [71]. Disruption of the circadian clock may interrupt cell growth, a biological process which is a risk factor for CRC [72]. The CREB-KDM4B-STAT3 signaling cascade is reported to be involved in DNA damage response in CRC [73], while cAMP signaling can promote CRC development disrupting normal apoptotic processes [74]. The endocannabinoid cancer inhibition pathway affects CRC cell growth [75], while the white adipose tissue browning signaling pathway discovered in this investigation is reported to play a major role in the molecular mechanisms linking obesity to CRC [76]. The dopamine receptor signaling pathway discovered here plays a major role in CRC [77,78]. For example, overexpression of dopamine receptor D2 (DRD2) has been shown to promote CRC progression by activating the β-catenin/ZEB1 axis [77]. It has been reported that elevated DRD2 expression is associated with a poor survival rate among patients with CRC [77]. This suggests that the dopamine receptor signaling pathway has potential to serve as a therapeutic target and that targeting the DRD2/β-catenin/ZEB1 signaling axis could potentially be a promising therapeutic strategy in clinical management of patients with CRC [77]. The ephrin receptor signaling pathway discovered in this investigation regulates autophagy [79]. High EphA2 receptor expression in CRC has been associated with worse outcomes in patients treated with cetuximab-based therapy [80], suggesting that the pathway has the potential to serve as a therapeutic target in CRC.

Other pathways discovered in this investigation associated with CRC included the glutamate receptor, axonal guidance, p53 and VEGF signaling pathways implicated in CRC [81,82,83,84,85,86]. The mGluR4 is overexpressed in CRC cells compared to normal colon cells [82,83]. The mGluR4 signaling pathway can enhance tumor cell proliferation and invasion, while overexpressed mGluR4 is correlated with a worse prognosis and poor disease-free survival [83]. The axon guidance signaling pathway is regulated by the netrin-1 receptor DCC(deleted in colorectal cancer), a single-pass transmembrane protein that belongs to the immunoglobulin superfamily, which has been implicated in CRC [84]. The P53 and VEGF signaling pathways are involved in multiple roles in CRC and constitute potential therapeutic targets [85,86]. Taken together, discoveries from this project and subsequent in silico validation suggest that DNA methylation has the promise for the discovery of potential therapeutic targets. Although we did not test the ability of the discovered molecular markers and pathways to serve as potential therapeutic targets, several studies have addressed that need for VEGF and P53 [85,86]. For example, Clark et al., 2022 have reported novel therapeutics that target key transcription factors in the DNA repair machinery involving P53 [86].

Overall, the clinical significance of these findings is that, because DNA methylation can be measured in body fluids such as urine and blood, if confirmed in such tissues, the CpG sites and genes discovered here could serve as noninvasive or minimally invasive molecular diagnostic and prognostic markers. Although this investigation was based on tumor samples, a number of studies have reported DNA methylation-based biomarkers with high sensitivity and specificity for blood-based and urine-based detection of CRC [87,88,89]. More importantly, because many current screening techniques use body fluids such as blood and stool [4,5] and DNA methylation could be easily measured in these fluids, DNA methylation-based biomarkers could be used to complement and or enhance current screening tools. Information on DNA methylation-based biomarkers could also be used in patient stratification in designing clinical trials, although this was not attempted here as it was beyond the scope of this investigation.

Limitations: Our investigation provides insights about the association of DNA methylation with CRC and demonstrates that CpG sites associated with CRC could predict survival outcomes. However, some limitations must be acknowledged. First, our discovery cohort consisted of patients with varying stages of CRC and some of them may have received postoperative radiotherapy with or without concurrent chemotherapy. In the absence of information on patient treatment, addressing that issue was beyond the scope of this investigation. However, although the identified CpG sites that were predictive of survival cannot be used for stratification of patients treated with intensive multimodal therapy, we believe that our findings may guide therapeutic decision making by providing clues to future de-escalation therapeutics. Secondly, our study used a limited sample size as opposed to all available samples in TCGA. This is because DNA methylation can be population-specific. While our discovered CpG sites and DMRs show very high associations of DNA methylation with CRC, these high associations may not be the norm as DNA methylation can be population- and tissue-specific.

## 5. Conclusions

Our investigation shows that DNA methylation-based molecular markers are associated with CRC, and have the promise to predict survival outcomes and discovery of potential therapeutic targets. New DNA methylation-based biomarkers that could aid in early CRC detection and prognosis detected by noninvasive and or minimally invasive methods are of critical importance to reducing the growing burden of CRC. Aberrant DNA methylation is an early event in CRC development and may be detected in body fluids and therefore constitutes a valuable CRC biomarker. Importantly, because DNA methylation is a stable epigenetic alteration that can be easily and rapidly quantified by methylation-specific PCR methods, it provides unprecedented opportunities for clinical implementation. Further research is recommended using large and diverse population cohorts to ensure that promising DNA methylation biomarkers undergo large-scale validation and their performance is compared with current screening techniques prior to their implementation in clinical practice. Future research will also focus on the tumor immune microenvironment.

## Figures and Tables

**Table 1:** List of the top 50 CpG sites that were most highly differentially methylated between tumor and control samples, and the genes and DNA methylated regions they map to.

Gene	Chromosome	CpG Site Probe	Feat.cgi	*p*-Value
*ADAMTS2*	5q35.3	cg14409941	Body-island	1.46 × 10^−33^
*ADAMTS5*	21q21.3	cg08190291	TSS200-island	2.21 × 10^−18^
*ADAMTS5*	21q21.3	cg21646598	TSS1500-island	1.46 × 10^−24^
*ADARB2*	10p15.3	cg02899206	TSS200-island	4.62 × 10^−30^
*ADARB2*	10p15.3	cg23684973	TSS1500-island	1.55 × 10^−29^
*ADCY1*	7p12.3	cg25322847	Body-shelf	1.13 × 10^−28^
*ADHFE1*	8q13.1	cg01588438	TSS200-island	1.07 × 10^−36^
*ADHFE1*	8q13.1	cg01988129	Body-island	6.84 × 10^−32^
*ADHFE1*	8q13.1	cg08090772	TSS200-island	8.19 × 10^−32^
*ADHFE1*	8q13.1	cg09383816	TSS200-island	7.76 × 10^−34^
*ADHFE1*	8q13.1	cg18065361	TSS200-island	1.68 × 10^−29^
*ADHFE1*	8q13.1	cg19283840	TSS200-island	2.53 × 10^−31^
*ADHFE1*	8q13.1	cg20295442	TSS200-island	3.59 × 10^−35^
*ADHFE1*	8q13.1	cg20912169	5′UTR-island	7.50 × 10^−35^
*ADRB3*	8p11.23	cg09258813	1stExon-island	3.29 × 10^−20^
*AGRN*	1p36.33	cg09248054	Body-island	7.98 × 10^−30^
*AGRN*	1p36.33	cg16318112	Body-island	2.59 × 10^−30^
*AHRR*	5p15.33	cg14453201	Body-island	1.07 × 10^−17^
*AKR1B1*	7q33	cg08167706	TSS1500-shore	1.86 × 10^−16^
*AMPH*	7p14.1	cg02383130	5′UTR-island	4.73 × 10^−20^
*AMPH*	7p14.1	cg07034660	Body-shore	1.10 × 10^−22^
*AMPH*	7p14.1	cg07926691	5′UTR-island	2.18 × 10^−27^
*AMPH*	7p14.1	cg10293925	5′UTR-island	2.30 × 10^−27^
*AMPH*	7p14.1	cg19875547	Body-island	8.40 × 10^−31^
*AMPH*	7p14.1	cg26122980	5′UTR-island	7.20 × 10^−30^
*ANK1*	8p11.21	cg17331296	1stExon-island	7.24 × 10^−28^
*ANKRD13B*	17q11.2	cg21101720	Body-island	2.94 × 10^−23^
*ANXA2*	15q22.2	cg22365276	5′UTR-shore	8.76 × 10^−26^
*AQP5*	12q13.12	cg26328335	TSS1500-island	4.63 × 10^−19^
*ATP11A*	13q34	cg08162124	Body-shore	1.02 × 10^−22^
*ATP8B2*	1q21.3	cg00581482	5′UTR-island	1.24 × 10^−22^
*ATP8B2*	1q21.3	cg08190044	5′UTR-island	1.72 × 10^−22^
*AUTS2*	7q11.22	cg21393713	1stExon-island	9.58 × 10^−32^
*AVP*	20p13	cg23035419	TSS1500-shore	4.05 × 10^−20^
*AVPR1A*	12q14.2	cg12516059	1stExon-shore	3.35 × 10^−23^
*B3GNTL1*	17q25.3	cg10344477	Body-shelf	1.50 × 10^−27^
*BARHL2*	1p22.2	cg26332310	1stExon-island	2.48 × 10^−19^
*BCAT1*	12p12.1	cg13980808	Body-shore	1.10 × 10^−18^
*BEND5*	1p33	cg11666087	1stExon-island	3.86 × 10^−19^
*BEND5*	1p33	cg16573178	1stExon-island	7.30 × 10^−17^
*BOLL*	2q33.1	cg03774803	1stExon-island	1.99 × 10^−25^
*BOLL*	2q33.1	cg13356896	TSS200-island	2.03 × 10^−28^
*BOLL*	2q33.1	cg24589459	TSS1500-island	4.46 × 10^−19^
*C9orf50*	9q34.11	cg09731694	1stExon-island	1.81 × 10^−32^
*C9orf50*	9q34.11	cg13405887	1stExon-island	4.58 × 10^−47^
*C9orf50*	9q34.11	cg14015706	1stExon-island	8.68 × 10^−39^
*CADM2*	3p12.1	cg05152589	5′UTR-island	1.17 × 10^−18^
*CALCR*	7q21.3	cg20276156	TSS1500-shore	2.43 × 10^−20^
*CASR*	3q13.33-q21.1	cg05937969	5′UTR-island	1.37 × 10^−22^
*CASR*	3q13.33-q21.1	cg25729826	5′UTR-island	9.43 × 10^−24^

**Table 2:** List of the 13 CpG sites that were most highly significantly differentially methylated between individuals who survived and individuals who succumbed to CRC (high indicates hypermethylated and low indicates hypomethylated).

Gene	Probe	Feat.cgi	*p*-Value	Methylated
*ADARB2*	cg02899206	TSS200-island	0.027	Low
*CDH12*	cg04480386	5′UTR-opensea	0.0068	Low
*DCLK1*	cg24239329	TSS200-island	0.011	Low
*DOK6*	cg27347269	TSS1500-island	0.037	Low
*EFS*	cg18844382	TSS200-island	0.038	Low
*GUCY1B3*	cg17493815	Body-island	0.035	Low
*KIAA1026*	cg00101629	Body-opensea	0.025	High
*MAGI2*	cg02523844	TSS1500-island	0.029	Low
*NKX2-2*	cg22474464	Body-island	0.023	Low
*NPBWR1*	cg24857620	TSS1500-island	0.0068	Low
*NR5A2*	cg03257172	Body-shore	0.01	Low
*PCSK2*	cg23875663	Body-shelf	0.045	High
*SMAD3*	cg24032190	Body-opensea	0.034	High

## Data Availability

All the original DNA methylation data and clinical information used in this investigation are publicly available in TCGA (https://www.cancer.gov/about-nci/organization/ccg/research/structural-genomics/tcga, accessed on 2 June 2022) and downloadable via the Genomics Data Commons https://gdc.cancer.gov/, accessed on 2 June 2022. Data generated from the analysis supporting the findings of this study are provided in supplementary tables in Supplementary Materials accompanying this report.

## Supplementary Materials

The following supporting information can be downloaded at: https://www.mdpi.com/article/10.3390/biomedicines10123163/s1, Supplementary Table S1: Complete list of all the CpG sites and genes associated with CRC. JWU; Supplementary Table S2: Complete list of all the 779 CpG sites and the 200 genes associated with CRC used in pathway analysis including CpG sites and genes predictive of survival. JWU; Supplementary Table S3: Complete list of all predicted gene regulatory networks enriched for CpG sites and functionally related genes associated with CRC. CH, DO; Supplementary Table S4. Complete list of all predicted signaling pathways enriched for CpG sites and functionally related genes associated with CRC. CH, DO.
